# Variations in coronary mortality rates between English primary care trusts: observational study 1993–2010

**DOI:** 10.1093/pubmed/fdv162

**Published:** 2015-11-06

**Authors:** L.S. Levene, R. Baker, K. Khunti, M.J.G. Bankart

**Affiliations:** 1 Department of Health Sciences, University of Leicester, 22-28 Princess Road W, Leicester LE1 6TP, UK; 2 Diabetes Research Centre, College of Medicine, Biological Sciences & Psychology, University of Leicester, Leicester Diabetes Centre, Leicester General Hospital, Gwendolen Road, Leicester LE5 4PW, UK

**Keywords:** coronary heart disease, morbidity and mortality, primary care, public health

## Abstract

**Background:**

In England, coronary heart disease (CHD) mortality has declined, but variations remain.

**Methods:**

This study aimed to describe under 75-year CHD mortality variations across geographically defined populations. Regression slopes for mortality data as a function of time were calculated for all 151 English primary care trusts (PCTs), giving the change in the expected age adjusted rate for each extra year.

**Results:**

Between 1993 and 2010, the mean age-standardized CHD mortality rate decreased from 107.76 to 35.12 per 100 000, but the coefficient of variation increased from 0.21 to 0.27. The slope of decline was significantly less after 2004 (*β* −4.91 for 1993–2003, −3.04 for 2004–2010). The proportion of smokers decreased by 24.6%. The estimated proportion of the population with controlled hypertension increased by 74.4% (2003–2010), but diabetes increased by 138% (1994–2010) and the proportion of obese people increased by 74.3% (1993–2010). There was a greater decline in CHD mortality in PCTs with greater deprivation and smoking (2006–2010).

**Conclusions:**

Since 2004, there has not been a relative reduction of variations in CHD mortality. Appropriate strategies to improve early detection and effective management of risk factors are needed to lower overall CHD mortality further and to reduce persistent variations across England.

## Introduction

Since the late 1970s, coronary heart disease (CHD) mortality rates have declined steadily in most industrialized countries,^1,2^ despite the rising prevalences of diabetes^3,4^ and obesity.^5^ Changes in risk factors (including reductions in total cholesterol, systolic blood pressure, smoking prevalence and physical inactivity) accounted for about half of the decline between 1980 and 2000,^6–8^ and just over one-third between 2000 and 2007, although this varied depending upon the level of socioeconomic deprivation.^9^

Populations across England have varying demographic and risk factor profiles.^10^ Between 1991 and 2007 geographical inequalities in all-cause mortality in under 75s increased in Britain.^11^ All-age CHD mortality rates varied geographically across England between 2006 and 2008, mostly explained by variations in population characteristics; however, greater detection of hypertension was associated with lower mortality.^12^

Since the 1990s, health policy in England has aimed to reduce CHD mortality rates. Pay for performance, the Quality and Outcomes Framework (QOF), was introduced into the general practitioner contract in April 2004, giving incentives to undertake more anticipatory and long-term condition care, including management of hypertension, diabetes, established CHD and hypercholesterolaemia. QOF has attracted international interest as a mechanism for driving quality in primary care.^13^ Since 1997, several initiatives have been introduced to reduce smoking, including a ban in workplaces and enclosed public places (in England since 2007), and the creation of National Health Service (NHS) stop smoking services to which patients can be referred.^14,15^

The aims of this paper are to describe trends and patterns of variation in CHD mortality across geographically defined population groups, to describe patterns in the variation of known cardiovascular risk factors, and to investigate whether variation and the rate of decline changed after the introduction of the QOF in 2004. We anticipated that the introduction of the QOF, with its incentives to improve care of conditions that influence CHD mortality, would be associated with a subsequent decline in CHD mortality.

## Methods

Premature mortality is death below an age limit. Under 75 years has been used as the definition of premature mortality by the Department of Health in England.^16^ Since ‘premature’ mortality is a public health priority,^17^ we focused on CHD mortality under the age of 75 years.

### Study design

An observational study was undertaken, involving primary care trusts (PCTs; geographically designated administrative bodies responsible for commissioning health services, replaced in 2013 by clinical commissioning groups) in England, in which mortality data are described for the years 1993–2010, the most recent year for which mortality data were published at this population level.

The population level of PCTs was studied rather than individual general practices, since we were unable to obtain mortality data at practice level, and reliable data for several population characteristics were not available at practice level. Repeated NHS re-organizations have meant that comparable datasets have been available for this population level only since 2006. Prior to 2006, national level data for population and service characteristics were used.

We supplemented a descriptive summary of CHD mortality trends for the whole period between 1993 and 2010 with a limited trend analysis comparing the periods before and after 2003.

The main sources of data for this study were the Health and Social Care Information Centre (HSCIC) (for mortality and service data), the Office for National Statistics (ONS) (for population data) and the Health Survey for England (HSE; some risk factor data for 1993–2010 at national level), unless otherwise stated.

### Study sample

The study included all PCTs in England. In 2006–2010, there were 152 PCTs. In 2010–2013, the number was 151, after two trusts merged. General practices contracted to PCTs delivered primary health care.

### Study variables

#### CHD mortality

Age-standardized mortality rates for CHD [International Classification of Diseases (ICD)-10 I20–I25 equivalent to ICD-9 410–414] in England from 1993 to 2010 at PCT population level for 0–74 years, 0–64 years and all ages were obtained from data on the HSCIC website.^18–20^ The ONS used postcodes to calculate mortality rates (based on death registers and population data) prior to 2006 in the PCTs that existed geographically in 2006–2010.

### Cardiovascular risk variables

The proportions of the English population aged 16–74 years estimated to be smokers, to be obese, to have hypertension, to have hypertension successfully treated <140/90 mmHg, and to have diabetes between 1993 and 2010 were calculated using data published by the HSE, a series of annual surveys designed to measure health and health-related behaviours in adults and children living in private households in England.^21–25^ These data were published with rates and sample size in age bands, enabling overall rates for these measurements to be calculated for 16–74 years.

### Demographic variables

The Index of Multiple Deprivation 2007 (IMD 2007) combined a set of indicators in seven domains (income, employment, health, education, housing, crime and environment) into a single score, and is the standard measure of socioeconomic deprivation in England. IMD scores at PCT level were obtained from the National Archives.^26,27^ Population estimates by ethnic group between 2002 and 2009 were obtained from the Office of National Statistics.^28,29^ These data were not age standardized.

### Primary healthcare variables

Selected QOF data (not age standardized) were obtained for the business years 2005–2006 to 2010–2011 (where available) for all 151 PCTs.^30–35^ We used numbers of registered patients (i.e. the number of patients registered with a practice), proportions of registered patients on hypertension and diabetes mellitus registers, proportions of hypertensive patients with controlled blood pressure and proportions of patients on obesity registers.

### Statistical analyses

The coefficient of variation (CV) was calculated for mortality rates. The CV of a measure is defined as the standard deviation divided by the mean. It thus aims to describe the variable's dispersion in a way not dependent on the variable's measurement unit.^36^ A rising CV suggests an increasing amount of variability relative to the mean over time, even if standard deviation values decline or remain constant. CVs have been used previously to describe trends in mortality variation.^37–39^

For 1993–2010, 0–74 years mortality data were modelled by creating linear regression slopes, calculated for each PCT, regressing age-adjusted mortality rates on years, with the result giving the change in the age-adjusted CHD mortality rate per extra year modelled. A segmented regression analysis was carried out in Stata version 12^40^ on the mean mortality rates to determine whether or not there was a change in slope comparing the period 1993–2003 with the period 2004–2010. In order to describe the pattern of results in terms of potential predictors between 2006 and 2010, quartiles were used to split each predictor into four equal-sized quarters, and within each quarter the mean slope for CHD mortality is presented. No formal statistical analyses were carried out associating CHD mortality with potential predictors because the potential predictors were not age adjusted, unlike the outcome. This incompatibility can lead to biased estimates.^41^

The other analyses were undertaken using SAS version 9.3.^42^

Ethical committee approval was not required, as only data already collected and publicly available were used. No individuals or practices were identified.

## Results

Between 1993 and 2010, the national CHD age-standardized mortality rate for 0–74 years decreased by two-thirds (from 107.76 to 35.12 per 100 000 European Standard Population^43^). The mean mortality of the directly standardized rate (DSR) for PCTs declined from 111.78 to 37.73, with the standard deviation declining from 24.20 to 10.25 (Table 1). The mean mortality for 0–64 years declined from 53.87 to 20.40 (standard deviation from 13.81 to 6.62) and for all ages from 203.67 to 77.30 (standard deviation from 33.49 to 15.23).

**Table 1:** Values of mortality rates and risk factor variables in England 1993–2010

*Year*	*0–74 years CHD mortality rate for England (age standardized per 100 000 European Standard Population)*	*Mean 0–74 years CHD mortality rates for 151 English PCTs (age standardized per 100 000 European Standard Population)*	*Standard deviation of 0–74 years CHD mortality rates for 151 English PCTs (age standardized per 100 000 European Standard Population)*	*Coefficient of variation (standard deviation/mean) of 0–74 years CHD mortality rates for 151 English PCTs*	*Estimated proportion in 16–74 years of smokers in England*	*Estimated proportion in 16–74 years of people with hypertension in England*	*Estimated proportion in 16–74 years of people with successfully treated hypertension in England*	*Proportion of treated patients (all ages) on CHD register with total cholesterol <5.0 mmol/l (90 mg/dl) (business year)*	*Estimated proportion in 16–74 years of obese people in England*	*Estimated proportion in 16–74 years of people with diabetes in England*
1993	107.76	111.78	24.20	0.2165	0.2850				0.1498	
1994	97.80	102.49	21.72	0.2119	0.2890				0.1567	0.0207
1995	94.07	99.04	21.07	0.2127	0.2925				0.1649	
1996	89.88	94.44	20.84	0.2206	0.3014				0·1741	
1997	83.78	88.35	20.53	0.2324	0.2927				0.1843	
1998	80.26	85.11	19.20	0.2256	0.2935				0.1948	0.0238
1999	74.12	78.43	17.76	0.2265	0.2838				0.2004	
2000	69.13	73.61	15.95	0.2167	0.2785				0.2121	
2001	65.09	69.62	16.78	0.2410	0.2706				0.2261	
2002	60.98	64.95	15.80	0.2432	0.2979				0.1918	
2003	57.20	61.69	15.26	0.2474	0.2706	0.2636	0.1290		0.2247	0.0335
2004	52.10	56.05	14.29	0.2549	0.2470				0.2316	
2005	48.29	51.98	13.09	0.2519	0.2461	0.3291	0.1998		0.2455	
2006	44.89	48.37	13.03	0.2694	0.2394	0.2517	0.1660	0.8192	0.2399	0.0419
2007	41.98	45.69	12.91	0.2826	0.2360	0.2599	0.1655	0.8251	0.2396	
2008	40.06	43.43	11.63	0.2677	0.2317	0.2591	0.1778	0.8028	0.2453	
2009	36.42	39.40	11.22	0.2847	0.2327	0.2531	0.1553	0.8026	0.2285	0.0452
2010	35.12	37.73	10.25	0.2718	0.2150	0.2589	0.2250	0.8210	0.2611	0.0496

Missing cells are where data were not available or could not be calculated.

**Fig. 1:**
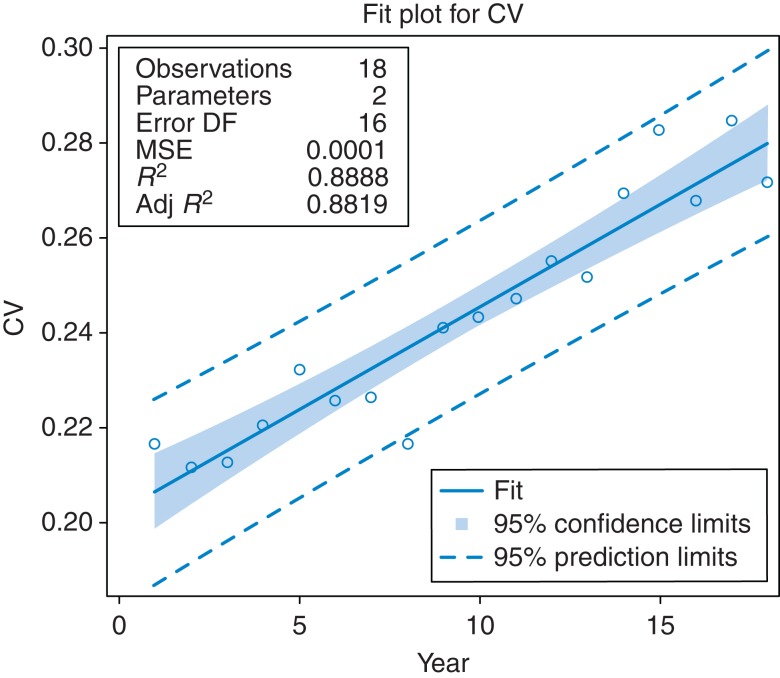
Fit plot for regression analysis of coefficient of variation.

For 0–74 years mortality, the overall trend of the CV increased from 1993 (0.2165) to 2010 (0.2718). A linear regression, regressing CV on year, was highly significant (*β* = 0.0043 [CI = 0.0035–0.0051, *P* < 0.0001]) (Table 2 and Fig. 1). Compared with 0–74 years, CV values were lower for all ages mortality (rising from 0.1644 in 1993 to 0.1970 in 2010) and higher for 0–64 years mortality (rising from 0.2564 in 1993 to 0.3244 in 2010). The CV values still rose after 2004 in all three age groups (starting at 0.3023 in 0–64 years, 0.2549 in 0–74 years and 0.1719 in all ages).

**Table 2:** Regression analysis of CV trend (regressing CV on year)

*Variable*	*DF*	*Parameter estimate*	*Standard error*	**t-*value*	*Pr > |*t*|*	*95% confidence limits*
*Parameter estimates*						
Intercept	1	0.20219	0.00413	48.95	<0.0001	0.19343–0.21095
Year	1	0.00432	0.00038162	11.31	<0.0001	0.00351–0.00513

Year 1 = 1993 and year 18 = 2010.

The rates for our calculations of the averages of these age-specific mortality rates were not weighted (by PCT population), because we intended each member of the sample to contribute equally to an analysis of variability. However, we recalculated the mean, standard deviation and CV after weighting each PCT's 0–74 years mortality rates by the 0–74 years population size (for 1993–2004 inclusive, we used the earliest available year's [2005] PCT population). The weighted means and standard deviations were slightly lower than the un-weighted values, but the effect on the trend was minimal.

The associations between year and (i) mean slope and (ii) standard deviation of the slope were approximately linear.

In the 16–74 years population, the proportion of smokers decreased by 24.6% in 1993–2010, the estimated proportion of the population with controlled hypertension increased from 12.9% in 2003 to 22.5% in 2010 (by 74·4%), the estimated proportion of people with diabetes increased from 2.1% in 1994 to 4.9% in 2010 (by 139%), and the estimated proportion of obese people increased from 15.0% in 1993 to 26.1% in 2010 (by 74.3%). The proportion of patients on a general practice CHD register with treated total cholesterol of 5.0 mmol/l (90 mg/dl) or less has remained consistently >80% since 2004 (Table 1).

The mean and standard deviation for the slopes of the PCTs' CHD mortality for 1993–2003 showed a bigger decline per year than for 2004–2010 (Table 3). After noting that the association between mortality and time for each of the two time periods, 1993–2003 and 2004–2010, appeared linear, separate linear regressions of the national mortality rate were conducted for the two periods, giving slopes of −4.89 [95% confidence interval (CI) −5.22, −4.56, *P* < 0.001] for the period 1993–2003, and −2.84 (95% CI −3.22, −2. 46, *P* < 0.001) for the period 2004–2010.

**Table 3:** Characteristics of the slopes for the PCTs' 0–74 years CHD mortality rates 1993–2010 (subdivided into 1993–2003 and 2004–2010)

*Period*	*1993–2003 with (95% CIs)*	*2004–2010 with (95% CIs)*
Mean of the slope	−4.87 (−5.13, −4.62) *P* < 0.0001	−2.87 (−3.24, −2.49) *P* < 0.0001
Standard deviation of the slope	−0.84 (−0.97, −0.71) *P* < 0.0001	−0.60 (−0.87, −0.33) *P* = 0.003
Coefficient of variation of the slope	0.0035 (0.002, 0.005) *P* = 0.0002	0.0037 (−0.003, 0.011) *P* = 0.22

Unstandardized *β* coefficients for slopes.

**Fig. 2:**
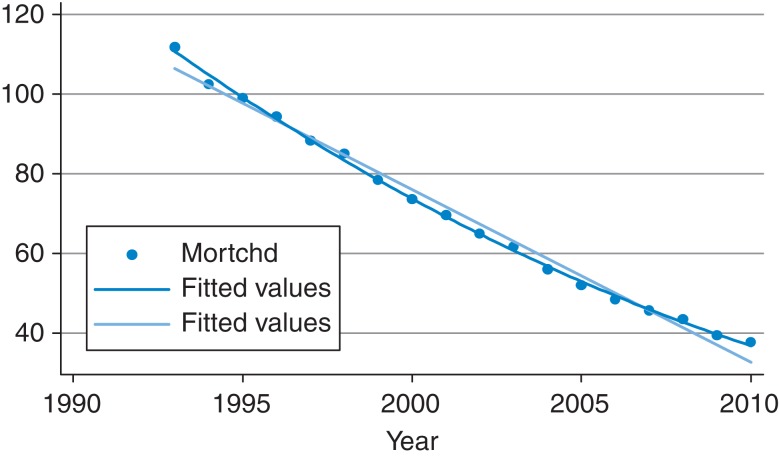
Graph showing linear and quadratic fits to the mean mortality (DSR) values by year (1993–2010). mortchd = age-standardized CHD mortality rate per 100 000 European Standard Population values. Vertical axis = age-standardized 0–74 years CHD mortality rate per 100 000 European Standard Population. Horizontal axis = year.

A quadratic term was then fitted, to get further evidence of a change in slope in national mortality rates over the entire period (1993–2010). The quadratic function (curvilinear line) appeared to be a closer fit to the data than the linear function (straight line), supplying further evidence that the slope changed at some point (Fig. 2). The quadratic term was highly significant (*P* < 0.001). A combined model was run, coding for separate slopes and intercepts for the two periods, 1993–2003 and 2004–2010, with time cantered around 2003. The similarity in the intercepts, 1993–2003 (*β* 50.65, 95% CI 48.84, 52.47, *P* < 0.001) and 2004–2010 (*β* 51.21, 95% CI 49.30, 53.13, *P* < 0.001) showed that there was no big jump in mortality between 2003 and 2004; however, there did appear to be a change in the slopes between the two periods. The lincom command^44^ was used to test the equality of the slopes generated for the two periods from the combined model. This produced a coefficient of 2.05 (95% CI 1.46, 2.65, *P* < 0.001) for the difference in slopes, *t* = 7.41, confirming a statistically significant difference between the two slopes.

For the period 2006–2010, the mean CHD mortality slopes by deprivation and smoking quarters were very similar. As deprivation scores or smoking rates increased, the mean slope became increasingly negative, suggesting that more deprived PCTs, and PCTs with more smokers might have had greater decreases in CHD mortality. This pattern was not observed for the other variables (obesity, hypertension register [detection], diabetes prevalence and non-white ethnicity), which appeared to have random univariable associations with CHD mortality (Table 4).

**Table 4:** Mean CHD mortality slopes (2006–2010) for variables by ascending quarter (PCTs divided into equal quarters by values for each variable—1 = lowest; 4 = highest values)

*Quarter*	*Mean slope*	*Standard deviation*	*Min*	*Max*
*IMD2007 score*				
1	−2.060514	0.9835176	−3.965	0.281
2	−2.347526	1.334425	−5.986	0.116
3	−2.825568	1.723624	−6.739	0.236
4	−3.779316	1.810079	−7.894	0.494
Total	−2.757367	1.624261	−7.894	0.494
*Smoking prevalence (estimated) 2006–2008*				
1	−2.076667	1.028924	−3.885	0.281
2	−2.278395	1.310488	−5.986	−0.542
3	−2.996769	1.766462	−7.894	0.236
4	−3.634395	1.778725	−7.035	0.494
Total	−2.757086	1.618841	−7.894	0.494
*Obesity prevalence (estimated) 2006–2008*				
1	−2.742861	1.607025	−7.035	0.281
2	−2.49827	1.750712	−6.824	0.494
3	−2.4215	1.337681	−5.192	0.236
4	−3.375816	1.652555	−7.894	−1.018
Total	−2.757086	1.618841	−7.894	0.494
*Diabetes practice register 2006–2007*				
1	−2.821946	1.475241	−5.658	0.281
2	−2.479711	1.150862	−6.654	−0.643
3	−2.964351	1.833222	−6.824	−0.051
4	−2.770605	1.945753	−7.894	0.494
Total	−2.757367	1.624261	−7.894	0.494
*Hypertension practice register 2006–2007*				
1	−3.30627	1.931512	−7.894	0.281
2	−2.976	1.584339	−6.824	0.116
3	−2.073946	1.125811	−3.979	0.494
4	−2.669711	1.557441	−6.739	−0.051
Total	−2.757367	1.624261	−7.894	0.494
*White ethnicity 2006*				
1	−3.027324	1.977129	−7.894	0.281
2	−3.023026	1.551053	−6.824	−0.489
3	−2.101514	1.090771	−5.997	−0.542
4	−2.867447	1.633851	−6.739	0.494
Total	−2.757367	1.624261	−7.894	0.494

For all ages, there was an upwards trend in both hypertension detection, from a mean/standard deviation of 12.4%/1.8% in 2006/07 to a mean/standard deviation of 13.6%/2.1% in 2011/12, and treatment success (of estimated population with hypertension), from a mean/standard deviation of 33.9%/2.7% in 2008 to a mean/standard deviation of 40.5%/3.3% in 2011.

## Discussion

### Main findings of this study

Despite the continuing decline in mean CHD mortality rates, wide variations remain between populations in England. The mean CHD mortality rates declined less quickly after 2005, but the CV remained static during that period, and was greater in the younger age group. A rise in CV suggests that a reduction in health inequality is unlikely to have occurred. Rates declined more in PCTs with higher levels of deprivation and with greater proportions of smokers, consistent with previous findings that the effect of changes in major cardiovascular risk factors upon CHD mortality varied depending upon the level of socioeconomic deprivation.^9^ Our findings are consistent with those of a recently published paper that appeared to show no association between QOF and premature mortality at practice level.^45^

The greater variation in mortality in the 0–64 years group, when compared with all age mortality, may possibly be explained by ‘survivors’ in the older age groups being less affected by variations in adverse risk factors. The longitudinal trend of geographical variations in premature CHD mortality in England was also consistent with that of premature all-cause mortality in the UK.^11^

### What is already known on this topic

Changes in risk factors accounted for about half of the decline between 1980 and 2000 in CHD mortality in England and just over one-third between 2000 and 2007^6^; although this varied, depending upon the level of socioeconomic deprivation. Trends in adverse risk factors have raised concerns about CHD mortality, particularly in young adults.^46^ There are substantial and persistent inequalities between European countries, with limited evidence that CHD mortality rates in younger age groups have been more likely to flatten than in older age groups.^47^ Attainment in QOF indicators has reached a sustained high level,^30–35^ but further improvements in cardiovascular disease have been achieved by better secondary care interventions and additional initiatives in primary care, such as local enhanced services and managed geographical practice networks.^48^ The adverse effects of rising levels of obesity and diabetes on CHD mortality may have been offset by smoking cessation, better control of hypertension, improving dyslipidaemia and possibly other factors. However, this offset may lessen in the future. Simple public health interventions, such as dietary salt restriction, have been shown to have the potential to reduce significantly cardiovascular events.^49^

Between 2003–2004 and 2011–2012, hospital care expenditure increased by 40% in real terms, compared with 22% in primary care.^50^ In the UK in 2009, of the total healthcare expenditure (£1.8 billion) for CHD, only 6% was in primary care and 15% was on medications.^51^

The greater investment in specialist care and the introduction of QOF into general practitioners' contracts fit into ‘an illness’ model of a health service, focusing more on delivering better care to those known to be ill or at increased risk, rather than on improving access to healthcare in whole populations. Although these have benefited the care of patients whose illness or risk have been diagnosed, greater specialization and the increasing use of single disease registers and care pathways may not address fully the increasing prevalence and the earlier onset of morbidity and multi-morbidity, particularly in more deprived populations.^52^ Tudor Hart observed that patients with the greatest health need often received the poorest-quality healthcare, known as the ‘inverse care law’.^53^ Starfield consistently found that increasing the supply of primary care results in lower heart disease mortality, after correcting for socioeconomic factors.^54^

Launched in 2009, but still under evaluation, NHS health checks aim to detect a range of diseases and cardiovascular risk factors in hitherto unscreened populations. The effectiveness of this scheme is as yet unclear. A parliamentary committee has recommended that the programme be scrutinized retrospectively by the UK National Screening Committee.^55^

Halting, and even reversing, rising rates of obesity and diabetes require not just medical interventions, but also initiatives that empower and encourage individuals to make healthier choices, whether through educational programmes, better support for increased physical activity, or clearer food labelling. In the financially stretched NHS, the balance of funding between primary and secondary care, and current public health policy priorities and strategies need review. A rigorous assessment of QOF's cost-effectiveness in relation to health outcomes has not yet been published.^56^ Increasing primary care's capacity to target and deliver cost-effective interventions to patients of low socioeconomic status with multiple morbidities has potential long-term public health benefits, but requires a sustained collaborative approach with greater capacity, such as suggested in the Scottish Deep End Project.^57^

### What this study adds

Despite a decline in mean CHD mortality rates, variations between geographical populations in England have persisted, and are greater in younger age groups. Rates declined more in PCTs with higher levels of deprivation and numbers of smokers.

### Limitations of the study

Our study relied on publicly available datasets, although there are limitations on the amount in the public domain. Reliable PCT population-level data for the relevant population and service variables were not available prior to 2006. In contrast to the mortality rates, none of the potential predictor rates was age standardized. Although there was some age banding of ethnicity numbers at PCT level, data for the proportion 0–74 years were not published at PCT population level. IMD scores were not age matched to the outcome.

HSE figures used samples of the population; the numbers were relatively small and CIs large, resulting in the calculated rates for some risk factors in a few years lying outside the longitudinal trends (see Table 1), but all of the HSE rates were for 16–74 years. Not having the full range of data at PCT population level after 2010 limited us to examining fully only a limited number of years.

Random fluctuations of rates in the smallest PCTs are unlikely to have a major effect on the calculation of the CV because these PCTs still have large populations and their rates were not outliers. However, CV values should be treated with caution as a measure of inequality, as different modelling assumptions and (non-CV) metrics might produce different answers.

In this analysis, the IMD was considered a stratifier with a fixed value during the period of study, and not as an independent variable in a classic multivariate regression.

## Funding

This work had no dedicated funding, but was supported by the Program of the National Institute for Health Research (NIHR) Collaboration for Leadership in Applied Research and Care (CLARHC) in Leicestershire, Northamptonshire, and Rutland and CLAHRC East Midlands. The funding organizations had no role in the design and conduct of the study, in the collection, analysis, and interpretation of the data, or in the preparation, review, or approval of the manuscript.

## Conflict of interest statements

K.K. reported being an advisor to the National Screening Committee and being a Clinical Advisor for the Diabetes NICE-led Quality and Outcomes Framework Panel. R.B. and K.K. hold NIHR Senior Investigator Awards, but the views expressed in this paper do not necessarily reflect those of the NIHR or of the Department of Health.
